# Newer Autoantibodies and Laboratory Assessments in Myositis

**DOI:** 10.1007/s11926-024-01171-8

**Published:** 2024-12-05

**Authors:** Georgina R Harvey, Christine MacFadyen, Sarah L Tansley

**Affiliations:** 1 Department of Life Sciences, University of Bath, Claverton Down, Bath, BA2 7AY UK; 2 Great Western Hospital, Swindon, UK; 3 Rheumatology, Royal National Hospital for Rheumatic Diseases, Royal United Hospitals Bath, Bath, UK

**Keywords:** Myositis, Idiopathic inflammatory myopathy, Autoantibody, Biomarker, Immunoassay, Detection

## Abstract

**Purpose of Review:**

We aim to describe the immunoassays that have been used for myositis autoantibody discovery with a focus on newer methods. We describe recently identified myositis autoantibodies that do not yet form part of routine clinical testing, highlighting what is known about their associated clinical phenotype and potential clues as to their presence.

**Recent Findings:**

Novel approaches to autoantibody detection have been employed in recent years including chemiluminescent immunoassay, phage immunoprecipitation-sequencing and modifications to the more traditional immunoprecipitation technique. This has led to the discovery of novel autoantibodies, including novel anti-aminoacyl-tRNA synthetase autoantibodies and autoantibodies which modify cancer risk for patients with anti-TIF1ɣ associated dermatomyositis.

**Summary:**

New approaches to novel autoantibody detection have facilitated autoantibody discovery and will enable the identification of autoantibodies to a broader range of autoantigens. Challenges remain in translating this knowledge into accessible testing particularly given the rarity of most recently discovered autoantibodies.

## Introduction

The idiopathic inflammatory myopathies (IIM), commonly referred to as “myositis”, comprise a heterogenous group of autoimmune diseases distinguished by proximal muscle weakness, cutaneous manifestations, and internal organ involvement. IIM can be subdivided according to clinical and pathologic features, into dermatomyositis (DM), polymyositis (PM), immune mediated necrotising myopathy (IMNM), anti-synthetase Syndrome (ASyS) and inclusion body myositis (IBM). Approximately 60–70% of patients with IIM have an identifiable myositis autoantibody [1–3]. Autoantibodies that are specific to myositis are termed myositis specific antibodies (MSA), whereas those which are also seen in other autoimmune connective tissue diseases are coined myositis associated antibodies (MAA). The detection of more than one MSA in the same individual is exceptionally rare [1], and as MSA correlate strongly with specific clinical phenotypes these are excellent biomarkers for identifying disease subgroups and the risk of key complications such as interstitial lung disease (ILD) and cancer. MSAs are routinely used biomarkers to facilitate IIM diagnosis and predict prognosis [4]. The clinical utility of MSA is demonstrated by their inclusion as part of risk-stratification within an international guideline for IIM-associated cancer screening [5].

The prevalence of autoantibodies in IIM is lower than in other connective tissue diseases such as Systemic sclerosis, Lupus and Sjogren’s syndrome where disease relevant autoantibodies can be found in > 80% patients. It seems likely that the 30–40% of IIM patients without an identifiable MSA or MAA have other autoantibodies yet to be discovered. In this article we describe the immunoassays that have been used for myositis autoantibody discovery and the myositis relevant autoantibodies that do not yet form part of routine clinical testing. We discuss what is known about the associated clinical phenotype of novel MSA and highlight potential clues as to their presence.

## Autoantibody Detection and Discovery Techniques

Immunoassays for the detection of MSAs and/or MAAs utilise a particularly varied set of biochemical and biophysical principles and applications. The essential features of some of these methods, specifically indirect immunofluorescence assay (IFA), enzyme-linked immunosorbent assay (ELISA), line immunoassay (LIA), dot immunoassay (DIA), addressable laser bead assay (ALBIA) and immunoprecipitation (IP), were described recently by Liu et al. [6]. in a narrative review. Additional immunoassay methods explained in recent research publications included RNA-IP [7], particle-based multi-analyte technology (PMAT) [8, 9], Western blotting (WB) [10], a new autoantibody array assay [11, 12], digital liquid chip method (DLCM) [13] and chemiluminescent immunoassay (CLIA) [13].

**Fig. 1:**
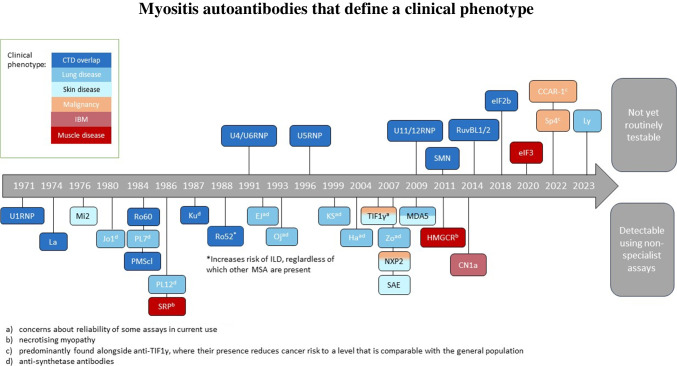
A range of different myositis specific and associated autoantibodies have now been described. Very rare autoantibodies and those discovered within the last 5–10 years are typically not included in standard testing panels. When patients test ‘negative’ for myositis autoantibodies clinicians should consider whether extended testing for CTD overlap autoantibodies e.g. U1RNP could be of benefit in addition to extended spectrum autoantibody testing for rare/novel autoantibodies, where facilities to do so are available

Many features vary between immunoassays: each has its own pros and cons which underly the applicability of each one to certain tasks and/or settings (Table 1). A relevant example here is the relative suitability of these assays to the discovery of novel autoantibody specificities in IIM and its overlap syndromes. Testing for MSA/MAA in patients with suspected IIM or overlap disease has become part of routine clinical care in most centres. Most immunoassays used in clinical practice for MSA/MAA detection are designed to detect a specific panel of autoantibodies and are not capable of identifying the presence of novel autoantibodies. Clinicians should be aware of those MSA/MAA which are detected by the assays available to them, in addition to those that are not included or may be missed due to poor assay performance [14–19]. Known MSA/MAA, their clinical associations and whether they can be detected by standard commercial assays is shown in Fig. 1.

**Table 1:** Immunoassays currently used for the detection and/or discovery of autoantibodies in the sera of patients with idiopathic inflammatory myopathies by (a) diagnostic and research settings, and (b) mainly research settings the traditional techniques of immunodiffusion and counterimmunoelectrophoresis are now uncommon. However, both WB and IP techniques are still widely used, particularly in research settings due to their high levels of sensitivity and specificity and their utility for the detection of novel autoantibodies. Developments in IP and WB include the use of IP-WB for the detection of scarce autoantigens, and the use of integrated systems for the identification of novel autoantigens using IP-MS. IFA continues to be popular for initial screening of sera in clinical settings, though it can also be used to help detect and identify novel autoantibodies in IIM. The last two decades have seen sustained growth in the use of multiplex diagnostic tests for diagnostic purposes in the clinical setting, as exemplified by LIA, DIA, CLIA, PMAT and ALBIA technologies. However, there are concerns regarding the sensitivity and specificity of some of these tests, particularly the LIA and DIA. Physicians should be aware that this may lead to a variable number of false positives and false negatives

Setting	Immunoassay	Discovery capability	Ease of use	Speed	Sensitivity	Specificity	Multiplex capability	Distinctive features	Specific problems
**(a) Diagnostic & research**	**IFA**	√	X	√	√	√	X	Can enable intracellular localisation of specific autoantigens; useful for screening	Subjective results; use of ICAP nomenclature recommended; no target identification
	**LIA**	X	√	√	Highly variable	X	√	Easy to use, convenient, and can be automated for rapid throughput; low equipment cost	False positives and false negatives are a major concern; costly reagents/consumables
	**DIA**	X	√	√	Highly variable	X	√	Easy to use, convenient, and can be automated for rapid throughput; low equipment cost	False positives and false negatives are a major concern; costly reagents/consumables
	**ELISA**	X	√	√	√	√	X	Cheap, easy, and efficient; high throughput; can be used quantitatively with standard curve; MSA detection reliable - often comparable to IP	Cross-reactivity; false positives may occur if non-specific blocking step is incomplete
	**CLIA**	X	√	√	√	√	X	Linearity of signal is improved relative to ELISA and sensitivity heightened; very rapid	Specialized equipment and reagents; expensive
	**PMAT**	X	√	√	√	√	√	Good agreement with protein IP; high throughput; quality controls for each analyte; quantitative and accurate	Specialized equipment and consumables; expensive
	**ALBIA**	X	√	√	√	√	√	Small serum samples are used; suitable for automation in a routine laboratory	Specialized equipment; can give false positives/negatives; costly
**(b) Mainly research**	**WB**	√	X	X	√	√	X	Enables identification of protein subunits containing an autoantigenic epitope	Protein denaturing: only capable of detecting linear epitopes
	**IP**	√	X	X	√	√	X	High level of specificity and sensitivity; can detect proteins with native epitopes; multi-enzyme complexes can be precipitated; regarded as ‘gold standard’ technique	Autoantigenic subunits are not apparent; safety concerns with use of [S-35] radioisotope; arduous technique
	**RNA-IP**	√	X	X	√	√	X	Enables identification of RNA components of RNP antigens, including those with native epitopes; multi-enzyme complexes can be precipitated	Avoidance of contamination by RNAse enzymes requires great vigilance; safety concerns with use of organic solvents
	**IP-WB**	√	X	X	√	√	X	Highly sensitive for detection of scarce autoantigens; enables identification of protein subunits containing an autoantigenic epitope	Protein denaturing: only capable of detecting linear epitopes if used as discovery technique
	**IP-MS**	√	X	X	√	√	X	Unparalleled for autoantibody discovery; useful for detection of rare autoantibodies	Mass spectrometry facilities are expensive and require expertise

*IFA* Indirect immunofluorescence, *LIA* line immunoassay, *DIA* dot immunoassay, *ELISA* enzyme-linked immunosorbent assay, *CLIA* chemiluminescent immunoassay, *PMAT* particle-based multi-analyte technology, *ALBIA* addressable laser bead immunoassay, *WB* Western blotting, *IP* immunoprecipitation, *RNA-IP* RNA immunoprecipitation, *IP-WB* immunoprecipitation-Western blotting, *IP-MS* immunoprecipitation-HPLC-tandem mass spectrometry, *MSA* myositis-specific autoantibodies, *RNP* ribonucleoprotein

### Immunoassays for Myositis Autoantibody Discovery

Immunoassays which enable autoantibody discovery share the straightforward practical characteristic of making the entire human proteome (or a large part of it) available and accessible to potentially novel autoantibodies contained in IIM patient sera. This has traditionally been achieved by preparing a whole-cell extract of human tissue-culture cells, typically K562 or HeLa cells, for incubation with patient autoantibodies immobilised onto a solid support, such as Protein-A Sepharose beads (as in IP), or by the fixation of tissue sections or tissue-culture cells onto the surface of a microscope slide, followed by incubation of the cells with diluted patient sera (e.g. the use of the HEp-2 cell line in IFA). Using such techniques, any ubiquitously expressed protein has the potential to be specifically targeted and bound by an IIM serum autoantibody which can then be detected using the appropriate assay-specific methods and compared with reference samples. It is important to be aware that a minority of MSAs recognise tissue-specific antigens, notably those found in muscle: this explains why standard IP, WB and IFA assays are unable to detect autoantibodies recognising the muscle-specific IIM autoantigen cN1A [20], and the requirement for separate testing of this specificity. In such cases, tissue-specific extracts can be made for IP or WB, as carried out during the original anti-cN1A autoantibody detection and identification studies by Pluk et al. [21].

A key feature of the WB technique, which also makes use of a whole-cell extract, is the complete transfer of all separated proteins from an SDS-PAGE gel to a nitrocellulose membrane. Only then is the membrane subsequently incubated with patient sera, allowing binding of autoantibodies to immobilised antigens, already separated according to molecular weight. By this stage, all proteins have been denatured by SDS-PAGE meaning that, while capable of autoantibody discovery, WB is limited to the detection of linear epitopes. By contrast, IP involves the incubation of patient autoantibody-coated Sepharose beads with whole-cell extract containing intact, native proteins. This enables binding of antigens to autoantibodies which recognise any of the surface features of correctly folded proteins or multienzyme complexes. Hence, any autoantigens have been selected before the denaturing electrophoretic stage and all their component polypeptides will be detected.

Once a novel autoantibody has been discovered, the unidentified autoantigen must then be characterised. In the past this may have involved immunoaffinity column chromatography of whole cell extracts followed by elution of any antigens bound by the immobilised autoantibodies and protein sequencing. However, in most cases, novel IIM autoantibodies have been identified by submission of the purified protein to a mass spectrometry (MS) service provider. There has been a concerted effort by some groups to use a pipeline approach to detect novel autoantibodies in the ~ 35% of IIM sera which have so far tested as seronegative. One such example is IP using unlabelled cell extracts immediately followed by HPLC-tandem MS (IP-MS) in the same laboratory, sometimes processing numerous samples in quick succession. This process was used to facilitate the recent discovery of the anti-Ly autoantibody by Vulsteke et al. [22]. The technique has the potential to revolutionise the detection of autoantibodies in IIM, at least in the research setting.

In an alternative novel approach, Hosono et al. applied Phage IP-Seq (PhIP-Seq) to an autoantibody discovery cohort of patients with DM negative for MSA following analysis with the EUROLINE Autoimmune Inflammatory Myopathies 16 Ag (IgG) test kit. PhIP-Seq identified patients with both anti-TIF1ɣ, which was ‘missed’ due to low sensitivity of the screening assay, and a novel autoantibody, anti-Sp4 [23].

The IFA assay using HEp-2 cells has long been used as a screening “ANA” test for a number of connective tissue diseases and determines the intracellular location of anti-cellular antibodies. The value of ANA-IFA as an autoantibody screening tool in IIM has been discounted by some, due to its low sensitivity for many MSA, the cytoplasmic location of aminoacyl tRNA synthetase (ARS) autoantigens, together with the tendency for further testing to concentrate on the anti-Jo-1 specificity [24]. However, it is hoped that the specific assignation of certain AC codes to cytoplasmic IFA patterns by the ICAP [25] will encourage the recognition of IIM sera which recognise cytoplasmic antigens: such an approach was exemplified recently by Yoshida et al. [26]. ANA pattern can thus provide a useful ‘clue’ as to the presence of an otherwise undetected MSA/MAA.

## Novel Autoantibodies Associated with Myositis Clinical Syndromes

### Anti-Synthetase Syndrome

ASyS is considered a distinct myositis clinical syndrome with features including myositis, interstitial lung disease, arthritis, Raynaud’s phenomenon, fever, and skin changes. Incomplete versions of the syndrome are recognised. The presence of an ASyS autoantibody is generally considered of key diagnostic importance but a validated set of classification criteria for ASyS remains outstanding. The ACR/EULAR CLASS (Classification Criteria of Anti-Synthetase Syndrome) project is a multi-national endeavour currently underway to address this [27]. There are twenty different ARS in humans, corresponding to the twenty different amino acids but thus far autoantibodies have only been described targeting approximately half of these: anti-Jo-1, targeting histidyl-tRNA synthetase, is the most common autoantibody in adults with IIM and can be identified in 15–30% of patients. The remaining anti-ARS autoantibodies are much rarer, collectively occurring in 10–20% of cases [1]. A key challenge appears to be that some patients with a compatible clinical phenotype for ASyS do not have an identifiable autoantibody. Though methods for the detection of autoantibodies are continuously evolving, clearly existing methods remain imperfect and not all testing platforms include the full range of known ASyS autoantibodies. Loganathan et al. used the CLASS project biobank to analyse methods for identifying ASyS antibodies in a large cohort of patients and controls and noticed differences in the reliability of real-world detection methods for different autoantibodies [28]. Specifically, methods for real-world detection of anti-Jo-1 antibodies were more reliable than for non-anti-Jo-1 antibodies, particularly anti-PL7 and rarer antibodies such as anti-OJ and anti-KS.

Of the known ASyS autoantibodies, anti-OJ uniquely targets an ARS which does not exist freely in the cytoplasm but forms part of a multi-enzyme complex containing several ARS and ribonucleoprotein subunits. Conformational epitopes and reaction with other ARS within this complex, in addition to isoleucyl-tRNA synthetase, have been demonstrated in some anti-OJ sera [29]. The PMAT assay allows simultaneous detection of autoantibodies recognising several different autoantigens. Using PMAT Fritzler et al. measured both anti-lysyl- and anti-isoleucyl-tRNA synthetase reactivities and concluded that such measurements may represent a method for the indirect measurement of anti-OJ autoantibodies [30].

Preger et al. [10] began from the premise that at least some seronegative IIM patients might have previously undetected anti-ARS autoantibodies. Therefore, using CLIA, each of multiple bead sets was coupled to a different specific recombinant ARS (both existing and potential IIM autoantigens were included) before incubation of the bead mixture with individual diluted patient sera. In their cohort of IIM patients, autoantibodies recognising all but three of the ARS were detected in at least one patient, representing nine novel ASyS autoantigens. Meanwhile, Sasai et al. [31] provided a case report concerning the detection of a novel specificity, anti-valyl-tRNA synthetase, in a patient with ASyS, and Vulsteke et al. used IP-MS to identify and characterise anti-cysteinyl-tRNA-synthetase reactivity, termed anti-Ly, in a patient with features of ASyS [22].

All anti-ARS autoantibodies produce cytoplasmic staining patterns on IFA, although these can be weak and may be missed at standard serum dilutions. Whilst not all laboratories will report cytoplasmic staining patterns on ANA testing, in the correct clinical context this can provide an important clue as to the presence of an otherwise undetected ASyS.

### Polymyositis

The term “polymyositis” has been dissected and it is now clear that distinct, more homogeneous patient subgroups can be described. These subtypes include IMNM, ASyS and IBM. Subsequently, the term “polymyositis” has largely been subsumed by these more specific diagnoses [32]. In 2020, Betteridge et al. described autoantibodies targeting eukaryotic initiation factor 3 (eIF3) in three adult patients with PM and reported prevalence of 0.4% in a European cohort [33]. None of the affected patients had a history of malignancy or ILD and all responded well to standard treatment. ANA analysis by IFA produced either a negative or weak, fine speckled ANA, and a fine cytoplasmic speckle. These could represent a very small group of patients where the label of PM still best applies.

### Overlap Myositis and Scleromyositis

Overlap myositis (OM) encompasses those patients with clinical and/or serological features of both IIM and other autoimmune connective tissue diseases. Systemic sclerosis (SSc) accounts for around 40% of cases and is the most common connective tissue disease seen in OM patients [34]. There is no currently accepted definition of Scleromyositis and, while meeting classification criteria for both diseases is often used in practice, this is insensitive. While there are overlapping clinical features, “Scleromyositis” should be considered a distinct syndrome and separate from both IIM and SSc. Myositis is a common first non-Raynaud’s symptom in this group and SSc skin involvement is often absent early in the disease course. Most patients with Scleromyositis have SSc specific or overlap autoantibodies, such as anti-PM/Scl, anti-Ku or anti-U1RNP. Rare autoantibodies, such as anti-RuvBL1/2, anti-U4/U6RNP, anti-U5RNP, anti-U11/U12RNP, anti-eIF2b and anti-SMN have also been described and a small proportion remain seronegative. Interestingly patients with ANA-negative Scleromyositis have been shown to be at higher risk for scleroderma renal crisis compared to ANA-positive patients. Anti-SMN reactivity appears to identify a particularly severe group with a higher rate of organ involvement, including ILD and Pulmonary Arterial Hypertension, in addition to features such as fingertip pitting scars, myositis, myocarditis and lower gastrointestinal involvement. A nuclear dots ANA pattern (ICAP AC6 or AC7) may help to identify those patients with anti-SMN autoantibodies in the absence of more specific testing methods. Patients with other rare autoantibodies (anti-RuvBL1/2, anti-U4/U6RNP and anti-U5RNP will often have a speckled ANA pattern (ICAP AC4 or AC5).

## Autoantibodies Which Modify Myositis Disease Phenotype

MAAs are antibodies that can be found in other connective tissue diseases (such as SLE, SS, SSc and MCTD) and may co-exist with an MSA. The additional finding of an MAA, alongside an MSA, can provide important additional clinical information. One example of this is anti-Ro52, which has been found to frequently co-exist with MSA and can identify patients at higher risk for ILD and poorer outcomes than those without anti-Ro52 [35].

### Novel Autoantibodies Which Modify Cancer Risk

It is now well established that some MSA are strongly linked to cancer associated myositis in adults and their utility in guiding cancer screening approach is demonstrated in the recent International Guideline for Idiopathic Inflammatory Myopathy-Associated Cancer Screening [5]. The most common ‘cancer-associated myositis’ autoantibody is anti-TIF1γ. While up to 50% of adult patients with this MSA have an associated malignancy, many patients never develop cancer [36]. Two novel autoantibodies, anti-Sp4 and anti-CCAR1, have recently been described which co-occur with anti-TIF1γ and significantly attenuate the cancer risk to a level comparable to that of the general population [23, 37, 38]. DM as a paraneoplastic syndrome is not fully understood but it is thought that the autoantibody response develops in response to cancer associated neoantigens to which the immune system is exposed via malignant cells. While anti-CCAR1 and anti-Sp4 appear to be the most common ‘cancer-modifier’ autoantibodies in anti-TIF1γ-positive DM, others have been described and an increasing diversity of the autoimmune response appears to be associated with a reduced likelihood of cancer emergence [37]. In the future these autoantibodies could form an important part of cancer risk stratification and screening strategies.

## Conclusions

MSA are established biomarkers used in routine clinical practice to aid diagnosis, inform prognosis and risk stratify IIM patients to identify those patients who would benefit from more thorough and/or frequent cancer screening. In the last few years, several novel autoantibodies have been identified, however except for anti-CCAR1 and anti-Sp4 which usually co-occur with anti-TIF1ɣ, novel MSA are typically very rare and found in a limited number of patients. ‘Seronegative’ thus remains the largest autoantibody defined IIM subgroup. Novel approaches to MSA discovery such as PhIP-Seq and modifications to more traditional techniques such as IP-MS facilitate the detection of a broader range of autoantigens and have aided the discovery of novel MSA. Consideration must also be given to how these and subsequent novel MSA will be detected in clinical practice; both in terms of assay sensitivity and specificity, but also accounting for a very low prevalence of rare MSA in populations being tested and the impact on positive predictive value.

## Data Availability

No datasets were generated or analysed during the current study.
